# Seroprevalence of Antibodies to Highly Pathogenic Avian Influenza A (H5N1) Virus among Close Contacts Exposed to H5N1 Cases, China, 2005–2008

**DOI:** 10.1371/journal.pone.0071765

**Published:** 2013-08-13

**Authors:** Qiaohong Liao, Tian Bai, Lei Zhou, Sirenda Vong, Junqiao Guo, Wei Lv, Libo Dong, Nijuan Xiang, Zi Li, Yang Huai, Jianfang Zhou, Xiaodan Li, Ray Y. Chen, Zhen Xu, Timothy M. Uyeki, Yuelong Shu, Hongjie Yu

**Affiliations:** 1 Key Laboratory of Surveillance and Early-warning on Infectious Disease, Division of Infectious Disease, Chinese Center for Disease Control and Prevention (China CDC), Beijing, China; 2 National Institute for Viral Disease Control and Prevention, Chinese Center for Disease Control and Prevention, Key Laboratory for Medical Virology, National Health and Family Planning Commission, Beijing, China; 3 Public Health Emergency Center, China CDC, Beijing, China; 4 World Health Organization (WHO) Beijing Office, Beijing, China; 5 Liaoning Center for Disease Control and Prevention, Shenyang, Liaoning, China; 6 Guangxi Center for Disease Control and Prevention, Nanning, Guangxi, China; 7 US Centers for Disease Control and Prevention’s International Emerging Infections Program, Beijing, China; 8 National Institute of Allergy and Infectious Diseases, National Institutes of Health, Bethesda, Maryland, United States of America; 9 Influenza Division, National Center for Immunization and Respiratory Diseases, Centers for Disease Control and Prevention, Atlanta, United States of America; The University of Hong Kong, Hong Kong

## Abstract

To assess the extent of highly pathogenic avian influenza (HPAI) A (H5N1) virus transmission, we conducted sero-epidemiologic studies among close contacts exposed to H5N1 cases in mainland China during 2005–2008. Blood specimens were collected from 87 household members and 332 social contacts of 23 H5N1 index cases for HPAI H5N1 serological testing by modified horse red-blood-cell hemagglutinin inhibition and microneutralization assays. All participants were interviewed with a standardized questionnaire to collect information about the use of personal protective equipment, illness symptoms, exposure to an H5N1 case during the infectious period, and poultry exposures. Two (2.3%) household contacts tested positive for HPAI H5N1 virus antibody, and all social contacts tested negative. Both seropositive cases had prolonged, unprotected, close contact with a different H5N1 index case, including days of bed-care or sleeping together during the index case’s infectious period, and did not develop any illness. None of the 419 close contacts used appropriate personal protective equipment including 17% who reported providing bedside care or having physical contact with an H5N1 case for at least 12 hours. Our findings suggest that HPAI H5N1 viruses that circulated among poultry in mainland China from 2005–2008 were not easily transmitted to close contacts of H5N1 cases.

## Introduction

Highly pathogenic avian influenza (HPAI) A (H5N1) viruses spread widely in poultry and migratory birds across 64 countries in Asia, Middle East, Europe and Africa [1], especially during 2005–06. During November 2003 to 16 June 2013, 630 human cases confirmed with HPAI H5N1 virus infection, including 375 deaths (60%) had been reported from 15 countries [2]. Most H5N1 patients have experienced severe pneumonia that often progresses rapidly to the acute respiratory distress syndrome [3]. Surveillance for H5N1 cases has mostly focused on hospitalized pneumonia cases, but the denominator of cases of human infection with HPAI H5N1 viruses, including asymptomatic [4] and mild illness [5], [6] is unknown. However, a meta-analysis inferred that a large number of people, particularly in Asia, have been infected with HPAI H5N1 viruses without severe illness [7].

Currently, sporadic human cases of HPAI H5N1 virus infection continue to be identified, especially in countries with enzootic HPAI H5N1 virus circulation among poultry. Recent experiments have demonstrated that genetically-modified HPAI H5N1 viruses were capable of respiratory transmission between ferrets [8], [9]. Furthermore, some of the mutations associated with transmission among ferrets are already present in HPAI H5N1 viruses currently circulating among poultry [10], [11].

The extent of avian-to-human and human-to-human transmission of HPAI H5N1 viruses should therefore be monitored through sero-epidemiological surveys, especially when symptomatic H5N1 cases are identified. Here we report the results of sero-epidemiologic studies conducted among close contacts exposed to H5N1 case-patients during 2005–2008 in mainland China.

## Materials and Methods

Between October 2005, when the first case of HPAI H5N1 virus infection was detected by surveillance in mainland China [12] and February 2008, 30 confirmed human H5N1 cases per WHO criteria [13] were identified. Of these H5N1 cases, 22 from southern China were infected by clade 2.3.4 H5N1 viruses, and one from northern China had clade 2.2 H5N1 virus infection. The clinical and epidemiologic characteristics of Chinese H5N1 cases have been reported elsewhere [14], [15]. We excluded the close contacts of seven H5N1 cases from the present analysis, as data were not complete for five cases’ contacts, and serological data from investigations of close contacts of two H5N1 cases in a family cluster were reported previously [16]. Therefore, we conducted sero-epidemiological investigations of antibodies to HPAI H5N1 viruses among the close contacts of 23 (77%) H5N1 cases from 11 provinces (Table 1).

**Table 1:** Survey sites, study population and antigens used in seroprevalence survey among close contacts exposed to HPAI H5N1 case-patients, China, 2005–2008.

Case-patientno.	Age (years),gender	Date ofillnessonset	Outcome	No. of close contacts registered for medical surveillance	No. of close contacts included in serological survey (%)	Survey sites	Antigens used	Clade of HPAI H5N1 virus	No. of seropositive cases in close contacts
1	9, M	Oct 10, 2005	Recovered	53	49 (92)	Xiangtan City,Hunan Province	A/CK/HN/21/05(H5N1)#	2.3.4	0
2	31, F	Oct 30, 2005	Recovered	7	6 (86)	Heishan County,Liaoning Province	A/CK/LN/23/05(H5N1)#	2.2	0
3	24, F	Nov 2, 2005	Died	16	15 (94)	Zongyang County,Anhui Province	A/Anhui/1/2005(H5N1)	2.3.4	0
4	35, F	Nov 11, 2005	Died	14	10 (71)	Xiuning County,Anhui Province	A/Anhui/2/2005(H5N1)	2.3.4	0
5	10, F	Nov 23, 2005	Died	71	56 (79)	Ziyuan County,Guangxi Province	A/Guangxi/1/2005(H5N1)	2.3.4	0
6	34, M	Dec 4, 2005	Died	15	13 (87)	Suichuan County,Jiangxi Province	A/Jiangxi/1/2005(H5N1)	2.3.4	0
7	6, M	Dec 17, 2005	Recovered	55	49 (89)	Guiyang County,Hunan Province	A/Anhui/1/2005(H5N1)*	2.3.4	0
8	35, F	Jan 3, 2006	Died	17	9 (53)	Jianyang City,Sichuan Province	A/Sichuan/1/2006(H5N1)	2.3.4	0
9	26, F	Jan 6, 2006	Recovered	16	11 (69)	Zhangpu County,Fujian Province	A/Anhui/1/2005(H5N1)*	2.3.4	0
10	29, F	Jan 10, 2006	Died	11	7 (64)	Chengdu City,Sichuan Province	A/Sichuan/2/2006(H5N1)	2.3.4	0
11	20, F	Jan 27, 2006	Died	14	9 (64)	Suining County,Hunan Province	A/Hunan/1/2006(H5N1)	2.3.4	0
12	9, F	Feb 10, 2006	Died	34	27 (79)	Anji County,Zhejiang Province	A/Zhejiang/1/2006(H5N1)	2.3.4	0
13	26, F	Feb 11, 2006	Recovered	8	7 (88)	Yingshang County,Anhui Province	A/Anhui/3/2006(H5N1)	2.3.4	1
14	32, M	Feb 22, 2006	Died	5	1 (20)	Guangzhou City,Guangdong Province	A/Guangdong/1/2006(H5N1)	2.3.4	0
15	29, F	Mar 13, 2006	Died	6	2 (33)	Shanghai	A/Shanghai/1/2006(H5N1)	2.3.4	0
16	21, M	Apr 1, 2006	Died	23	16 (70)	Wuhan City,Hubei Province	A/Hubei/1/2006(H5N1)	2.3.4	0
17	8, F	Apr 16, 2006	Recovered	77	61 (79)	Suining City,Sichuan Province	A/Sichuan/3/2006(H5N1)	2.3.4	0
18	37, M	Dec 10, 2006	Recovered	24	20 (83)	Huangshan City,Anhui Province	A/Anhui/1/2005(H5N1)*	2.3.4	0
19	44, F	Feb 18, 2007	Recovered	10	9 (90)	Jianou City,Fujian Province	A/Fujian/1/2007(H5N1)	2.3.4	0
20	16, M	Mar 17, 2007	Died	10	8 (80)	Bengbu City,Anhui Province	A/Anhui/4/2007(H5N1)	2.3.4	0
21	22, M	Jan 16, 2008	Died	9	9 (100)	Jianghua County,Hunan Province	A/Hunan/1/2008(H5N1)	2.3.4	1
22	41, M	Feb 12, 2008	Died	12	7 (58)	Nanning City,Guangxi Province	A/Guangxi/1/2008(H5N1)	2.3.4	0
23	44, F	Feb 16, 2008	Died	20	18 (90)	Haifeng County,Guangdong Province	A/Guangdong/1/2008(H5N1)	2.3.4	0
Total	–	–	–	527	419 (80)	–			2

# A/CK/HN/21/05(H5N1) and A/CK/LN/23/05(H5N1) isolated from poultry epidemiologically linked to case-patient 1 and case-patient 2 respectively, were used as antigens in the serological assays for close contact exposed to the matched H5N1 case; these two patients were confirmed by serology only.

* For serological testing of exposed contacts without isolation of H5N1 viruses from either human cases or poultry epidemiologically linked to cases, a representative H5N1 virus strain [clade 2.3.4, A/Anhui/1/2005(H5N1)] was used as the antigen.

### Definitions

Close contacts, including household and social contacts, were defined as individuals who reported face-to-face contact within 1 meter of an H5N1 case, or direct contact with an H5N1 case-patients’ respiratory secretions or feces, or clothing contaminated with respiratory secretions or feces, during an H5N1 case-patients’ infectious period. The infectious period was defined as the time beginning one day before the illness onset of an H5N1 case-patient to the time of hospital discharge or death. Household contacts were defined as all persons who lived with an H5N1 case for part or all of the case-patient’s infectious period. Social contacts were defined as non-household contacts and included relatives, visitors, neighbors, colleague, teachers, classmates, roommates, friends, and others. Serology results were included if contacts’ convalescent sera were collected ≥11 days (minimum incubation period of 3 days in clusters in which human-to-human transmission might occur [3] plus minimum time for antibody response 8 days after illness onset [17]) after the last exposure to the corresponding H5N1 case.

### Enrollment

Once an H5N1 case was confirmed, an investigation team including staff of the local CDC and China CDC were immediately sent to investigate any potential source of HPAI H5N1 virus infection for the index case and identify his/her close contacts for additional case finding, and to assess the potential for human-to-human transmission. If the investigators identified households and places (e.g. poultry farm, wet poultry market, restaurant, health care facilities, work place) known to have been visited by the H5N1 index case during their infectious period, all persons in the household and at places visited by an index case were screened to identify close contacts. As part of the public health outbreak response to H5N1 in China, close contacts are required to be registered and placed under daily medical surveillance for fever and respiratory symptoms for 10 days after their last exposure to an H5N1 case. These contacts were advised to limit movements and stay home during this period.

Informed consent was obtained from participants during their 10-day quarantine following exposure to a confirmed H5N1 case. Eligible study subjects consisted of adults or children aged >1 year, and who met the definition of a close contact.

### Questionnaire

A structured questionnaire was developed and administered to each participant during a face-to-face interview after obtaining consent to participate in the study. We collected information on demographics, use of personal protective equipment, use and compliance with antiviral chemoprophylaxis, influenza vaccination status, illness symptoms, exposure to an H5N1 case, and other potential H5N1 risk factors (direct or close contact with sick, dead, or well-appearing poultry, visiting a wet poultry market, and exposure to individuals with febrile respiratory illness) during the period starting two weeks before the last exposure to an H5N1 index case up to the time of questionnaire administration. An adult household member (e.g., parent or legal guardian) was interviewed as a proxy for any study participant contact who was considered incapacitated or aged <10 years old.

### Specimen Collection

A blood specimen was collected from close contacts for acute and convalescent sera (≤1 week, and ≥11 days after the last known exposure to an H5N1 case, respectively) for H5N1 serological testing. Each blood specimen was collected by venipuncture (5 ml for those ≥18 years old; 2–5 ml for those 10–17 years old; 1–2 ml for those <10 years old), and placed on ice packs, and transported to the local CDC for processing. Serum was separated, split into 4 aliquots and temporarily frozen at −20°C at local CDC laboratories, and shipped on dry ice to the National Influenza Center (NIC) in Beijing, Chinese Center for Disease Control and Prevention (China CDC) within 4 days after collection and stored at −70°C.

### Laboratory Testing

All sera were tested between March and July 2008 at the NIC. Appropriate antigens for the serological assays were selected based on the antigenic characteristics of HPAI H5N1 viruses circulating in the region at the time of sera collection. We used HPAI H5N1 virus strains isolated from 18 of 23 H5N1 cases, and two viruses isolated from poultry that were epidemiologically-linked to two serologically-confirmed H5N1 cases as antigens in the serological assays for each contact exposed to the corresponding H5N1 case (case-patient numbers 1 and 2, Table 1). For serological testing of exposed contacts of three H5N1 cases (case-patient numbers 7, 9, 18) from southern China without isolation of HPAI H5N1 viruses from either the respective H5N1 index cases or poultry epidemiologically-linked to the cases, a representative HPAI H5N1 virus strain [clade 2.3.4, A/Anhui/1/2005(H5N1)] was used as antigens.

We conducted antigenic analysis by testing three HPAI H5N1 index case-patients’ convalescent sera (case-patient numbers 2, 7, 18) against the strains which were selected as antigens to test the sera for their contacts. We found that the selected strains were antigenically similar. A four-fold increase of antibody against HPAI H5N1 virus was found when we used A/CK/HN/21/05(H5N1) [18] and A/Anhui/1/2005(H5N1) to test the acute and convalescent sera for two HPAI H5N1 index cases (case-patient numbers 1, 9), respectively. Thus we believe that antigenically similar HPAI H5N1 virus strains were used to test the sera for contacts of these five index cases from whom HPAI H5N1 virus strains were not isolated.

All samples were screened using the modified horse red-blood-cell hemagglutinin inhibition (HI) assay [19] for H5N1 virus antibodies in bio-safety level (BSL) 2 conditions. Sera with an HI titer of ≥40 were then tested by microneutralization (MN) [20] assay in an enhanced BSL 3 containment laboratory. The HI assay can detect antibody against the globular head of the hemagglutinin, and a broadly neutralizing anti-HA stalk antibody produced by HPAI H5N1 virus infection can be detected by the MN assay alone. [21], [22] Because of limited resources, we only tested a random sample of sera with an HI titer of <40 for neutralizing antibody using MN assay for quality control.

Sera with a neutralizing antibody titer ≥40 and an HI antibody titer ≥40 were adsorbed with circulating seasonal influenza A (H1N1) and A (H3N2) viruses at that time of specimen collection to reduce the possibility of detecting antibodies that were cross-reactive to human influenza A viruses. Red blood cells (RBCs) were pre-treated by an equivalent volume of potassium periodate (KIO4) with a very low concentration of 0.5 mmol/L for 15 minutes at room temperature [23]. The treated RBCs were then adsorbed by seasonal influenza viruses with a concentration of 6000 hemagglutinin units for 10 minutes at room temperature. Residual virus was washed by phosphate buffer saline twice by centrifugation. The packed RBC-Virus mixture was then equivalently adsorbed with sera for 2 hours at room temperature. The RBC-Virus mixture was removed by centrifugation and sera were transferred to new tubes for use. The MN test was then repeated. No change in HPAI H5N1 virus antibody titer after adsorption indicated the presence of anti-H5 antibody, while a >4-fold reduction in MN titer after adsorption was interpreted as evidence for significant cross-reactivity. Sera were tested in duplicate by two separate MN assays performed on different days.

An individual was defined to be seropositive for HPAI H5N1 virus antibody for the purposes of this study modified from WHO criteria [13], [19]: (1) For single serum, an HPAI H5N1 virus neutralizing antibody titer of ≥40 for study subjects aged ≤14 years old, or ≥80 for those aged 15–59 years old, with an HI titer of ≥40; or (2) a four-fold or greater rise in neutralizing antibody titer against HPAI H5N1 virus in paired acute and convalescent sera, with the convalescent serum having a neutralizing antibody titer of ≥80 for adults or ≥40 for children or an HI titer of ≥40.

Adults aged ≥60 years were excluded from analysis because of decreased specificity of the MN assays for this age group [24]. The neutralizing antibody titers and HI titers are expressed as the mean of 2 determinations and undetectable titers of <10 are expressed as 5.

### Statistical Analysis

Data were entered in duplicate and verified using EpiData software (Odense, Denmark, accessed at: http://www.epidata.dk/links.htm). Data were analyzed with SPSS (version 17.0, SPSS Inc., Chicago, Illinois, USA). Median and interquartile range were calculated for continuous variables, and proportions were calculated for categorical variables. Ninety-five percent confidence intervals for seroprevalence were estimated with Poisson methods.

### Ethics Statement

The study was approved by the Institutional Review Board of China CDC in April 2007. During the early stage of HPAI H5N1 outbreak before April 2007 in China, this study was considered to be part of a continuing public health outbreak investigation by National Health and Family Planning Commission of China and exempt from institutional review board assessment. Therefore, written informed consents were not obtained from the study subjects who were enrolled before approval of this study. The Institutional Review Board of China CDC waived the need for written informed consent from those participants and agreed that we anonymized their specimens and personal information by permanently removing personal identifiers from the database. Anonymized samples were relabeled with a new random coding system. After April 2007, we requested participation upon written informed consent from adults or a parent or legal guardian for minors aged <18 years and informed assent from study subjects aged 10–17 years.

## Results

From October 2005 through February 2008, 527 close contacts of 23 H5N1 cases were identified. Of these, we enrolled 419 (80%) including 87 household members and 332 social contacts. The median number of close contacts that were enrolled per H5N1 case was 10 (range:1–61). Household contacts mainly consisted of family members, except four roommates of one patient who lived with their colleague.

### Demographics and Underlying Risk Conditions

The median age of the 419 close contacts was 19 years (range: 2–59 years), and 51% were male. Household members were significantly older than social contacts (32 years vs. 11 years, p<0.05). Other demographic characteristics and underlying risk conditions were similar between the two groups of contacts (Table 2). Of 71 contacts with available information, six (8%) had underlying medical conditions, including five with neurological disease, and one with a haemangioma. Only 2% (5/321) of close contacts reported receiving seasonal influenza vaccination within the previous year.

**Table 2:** Demographic characteristics, exposure history, use of personal protective equipment, and serum collection of 419 close contacts exposed to HPAI H5N1 case-patients, China, 2005–2008.

	Household contacts,N = 87, n (%)	Social contacts,N = 332, n (%)	Total, N = 419, n (%)
Median age – (years [interquartile range])	32 [17]–[43]	11 [9]–[41]	19 [9]–[41]
Male	42 (48)	172 (52)	214 (51)
Underlying medical conditions	1/17 (6)	5/54 (9)	6/71 (8)
Seasonal influenza vaccination within previous year	0/61 (0)	5/260 (2)	5/321 (2)
Sera sample collection			
Provided single serum specimen	52 (60)	136 (41)	188 (45)
Median duration between last exposure to H5N1 patient and serumcollection-(days [interquartile range])	43 [29–70]	29 [17–72]	33[18–71]
Provided paired acute and convalescent sera	35 (40)	196 (59)	231 (55)
Median duration between last exposure to H5N1 patient and acute serum collection- (days [interquartile range])	4 [2]–[7]	6 [6]–[7]	6 [6]–[7]
Median duration between last exposure to H5N1 patient and convalescentserum collection- (days [interquartile range])	57 [30–89]	54 [39–136]	54 [38–98]
Exposed to H5N1 case-patients			
Provided bedside care	26 (30)	25 (8)	51 (12)
Median duration of exposure- (hours [interquartile range])	108 [28–233]	10 [2]–[28]	28 [5–140]
Only had physical contact	61 (70)	1 (0)	62 (15)
Median duration of exposure- (hours [interquartile range])	35 [4–120]	6 [6–6]	30 [4–120]
Only had indirect contact within 1 meter	0 (0)	306 (92)	306 (73)
Median duration of exposure- (hours [interquartile range])	–	3 [1]–[12]	3 [1]–[12]
Personal protection equipment (PPE) while exposed to H5N1 case-patients			
N95 respirator	0 (0)	0 (0)	0 (0)
Surgical mask	4 (5)	8 (2)	12 (3)
Goggle	0 (0)	0 (0)	0 (0)
Face shield	2 (2)	0 (0)	2 (0)
Gloves	3 (3)	3 (1)	6 (1)
Gowns	3 (3)	1 (0.3)	4 (1)
Exposed to poultry			
Contact with well-appearing poultry#	51 (59)	159 (48)	210 (50)
Contact with sick or dead poultry#	17 (20)	35 (11)	52 (12)
Visited wet poultry market*	5/17 (29)	17/54 (31)	22/71 (31)
Antiviral chemoprophylaxis			
Oseltamivir	0 (0)	0 (0)	0 (0)
Other antivirals	26 (30)	46 (14)	72 (17)
With febrile respiratory illness	1 (1)	4 (1)	5 (1)

Data are no. (%) of close contacts, unless otherwise indicated. Percentages may not total 100 because of rounding.

# Contact with well-appearing or sick/dead poultry was defined as direct contact (e.g. touching), or indirect contact which was defined as no physical contact, but being within 1 meter of poultry, poultry products, or poultry feces.

* A wet poultry market was defined as a place where small animals and poultry may be purchased live or slaughtered at the market.

### Serology Results

**Figure 1:**
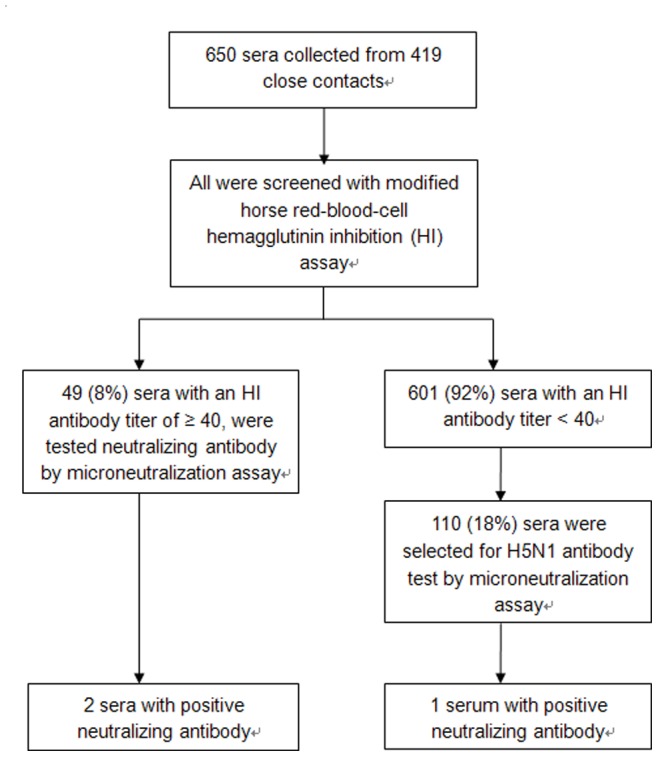
Flowchart of H5N1 serological testing for 419 close contacts exposed to HPAI H5N1 cases, China, 2005–2008.

A total of 650 sera were collected from 419 close contacts, including 188 (45%) with a single serum specimen and 231 (55%) with paired acute and convalescent sera (timeline of serum collection shown in Table 2). Screening by HI assay yielded antibody titers of 40 in 33 (5%) sera and ≥80 in 16 (2%) sera. The distribution of HI antibody titers in contacts for whom both acute and convalescent sera were available are shown in Table S1. All positive samples with an HI titer ≥40 were tested by MN assay, in addition to 110 sera randomly selected with HI titers <40 (Figure 1). Interestingly, serum from an 11-year-old boy collected 21 days after his last exposure to the first reported H5N1 case in November 2005, tested positive for HPAI H5N1 virus antibody (MN titer = 40), although his HI titer was 20. He was a classmate of the index H5N1 case (case-patient number 1, Table 1) and sat close by the case for 5 days after the case-patient’s illness onset.

Overall, only two (0.48%, 95% confidence interval: 0.05%–1.72%) of the 419 close contacts [2 of 188 (1.06%, 95% confidence interval: 0.11%–3.83%) with a single convalescent serum specimen collected] had serum that met our definition as testing seropositive for HPAI H5N1 virus antibodies. No change in HPAI H5N1 virus antibody titer was found for these two close contacts after adsorption with human influenza A human influenza A (H1N1) and A (H3N2) viruses. Both seropositive individuals lived with H5N1 cases, yielding an HPAI H5N1 virus antibody seroprevalence of 2.3% (2/87, 95% confidence interval: 0.2%–8.3%) among household contacts. Neither of these two contacts developed fever or any respiratory symptom during the post-exposure medical surveillance period. Of the 231 close contacts with paired sera, no evidence of sero-conversion was detected. All 332 social contacts tested seronegative for HPAI H5N1 virus antibodies.

The first seropositive case was the 4-year old daughter of an H5N1 case (case-patient number 13, Table 1) that survived. A single serum specimen was collected from the child on day 63 after the last known exposure to the index case (her mother); this serum had a neutralizing antibody titer of 40 and an HI titer of 80. The child slept with her mother and had unprotected direct and close physical contact with her for 5 days from the mother’s illness onset on 11 February 2006 to her hospital admission date. During this period, the mother experienced high fever and productive cough. In addition, four other household contacts and two social contacts of the index H5N1 case were also enrolled into this study. The four household contacts shared meals together with the index case for 5 days during the infectious period; however, none reported providing bedside care or direct physical contact with her. The two social contacts reported close contact (within one meter without physical contact) for<1 hour with the index case. All close contacts did not wear any protective equipment during their exposure to the index case. Poultry were raised in the backyard of the index case’s household. Six days before the index case’s illness onset, 13 chickens were found to be sick and later died quickly. The chickens were buried by the grandfather of the 4-year old seropositive child. There was no report of any direct contact between the seropositive case and the sick or dead chickens. All other close contacts were seronegative for HPAI H5N1 virus antibodies.

The second seropositive case was the 43-year old father of an H5N1 case (case-patient number: 21). A neutralizing antibody titer of 120 and an HI titer of 80 were detected in serum collected on Day 24 after the last exposure to his 22-year-old ill son. The father had close contact with his ill son for 9 days after the son’s illness onset on 16 January 2008 until he died. The father provided unprotected bedside care when his son was sick with HPAI H5N1 virus infection. He did not report contact with any other ill persons. We also enrolled two other household contacts (the index case’s mother and sister) and six social contacts, and all of them were seronegative for HPAI H5N1 virus antibodies. The index case’s mother also cared for him for 9 days, and his younger sister cared for him for one day, but neither wore any protective equipment. Of six social contacts that visited with the index patient within one meter (without direct contact), five visited for less than one hour, but one contact (the case’s uncle) was present in the home for 7 days. Three days before the index patient’ illness onset, chickens in the neighbor’s household began to die and all were dead within three days. Five chickens in the index case’s household began to die on two days after the index case’ illness onset and all were dead within two days. The father did not have direct or indirect contact with dead poultry. All the dead chickens were buried by the index patient’s mother.

### Exposure History and Antiviral Chemoprophylaxis

The proportions of close contacts that provided bedside care, had physical contact, or reported close, but not direct contact within one meter (indirect contact) of an H5N1 index case were 12% (n = 51), 15% (n = 62), and 73% (n = 306), respectively. Only three contacts had direct contact with case-patients’ respiratory or fecal secretions, but all were seronegative for HPAI H5N1 virus antibodies. Among all contacts, 71(17%) reported direct contact (provided bedside care or had physical contact) with H5N1 cases for at least 12 hours. None of the 419 contacts wore appropriate personal protective equipment when exposed to H5N1 cases during the case-patients’ infectious period, and only very few reported wearing a surgical mask (3%, n = 12), gloves (1%, n = 6), or gown (1%, n = 4). Twelve-percent of close contacts were also exposed to sick or dead poultry, and 31% (22/71) had recently visited a wet poultry market. The exposure history of household contacts and social contacts is summarized in Table 2. Household contacts were more likely to provide direct contact with H5N1 patients than social contacts (100% vs. 8%, p<0.05), and had longer duration of exposure (median: 56 hours vs. 8 hours, p<0.05). Five contacts developed mild febrile respiratory illness, however, none tested positive for HPAI H5N1 virus antibodies by MN assay after a median 29 days following last exposure to the index cases.

None of the 419 close contacts received oseltamivir for chemoprophylaxis. However, close contacts of three H5N1 cases (n = 72, 17%) were given other antivirals for chemoprophylaxis against influenza. This included 49 contacts of an H5N1 case that received moroxydine for 3 days (100 mg, by mouth, three times daily), 7 contacts of an H5N1 case that received rimantadine for 3 days (50 mg, by mouth, twice daily), and 16 contacts of an H5N1 case that received amantadine for 5 days (100 mg, by mouth, twice daily). None of the contacts who received any antiviral chemoprophylaxis tested positive by both HI and MN assays.

## Discussion

Our findings suggest that HPAI H5N1 viruses that circulated among poultry in mainland China during 2005 to 2008 were not easily transmitted to or among humans. Among all 419 household and social contacts of H5N1 cases with at least a convalescent serum specimen, the seroprevalence of HPAI H5N1 virus antibodies was 0.48%, including 2.3% among 87 household contacts, and 0% among 332 social contacts. The two seropositive household contacts were asymptomatic. None of the 419 close contacts reported using appropriate personal protective equipment, and 17% reported providing bedside care or having physical contact with an H5N1 case for at least 12 hours.

Our findings are consistent with other published data on HPAI H5N1 viruses that circulated among poultry during the same periods [25]–[29]. In 2005–2007, sero-epidemiological surveys reported 0–3% seroprevalence of H5 antibodies in populations exposed to infected poultry and in poultry workers. Few seroprevalence data among household contacts have been published since 2004. One study conducted during the 1997 Hong Kong outbreak [30] suggested a higher frequency of HPAI H5N1 virus transmission: 6 (12%) of 51 households contacts were seropositive, but none of 26 social contacts. Differences in these results may be attributable to variability in exposures, host factors, or differences in the adaptability of HPAI H5N1 viruses to humans [31]. Other sero-epidemiologic studies in which transmission to close contacts of confirmed H5N1 cases was evaluated after 2004 [16], [32]–[34] have found no evidence of human-to-human HPAI H5N1 virus transmission. However, most of these studies have assessed the potential for nosocomial transmission from H5N1 case-patients to healthcare workers, except for a very limited study in household and social contacts in China [16].

Due to the few seropositive cases identified, we were unable to study possible modes of transmission or risk factors for HPAI H5N1 virus infection. It has been suggested that HPAI H5N1 virus transmission to humans occurs through inhalation, ingestion, or nasal or conjunctival inoculation of material contaminated by HPAI H5N1 virus [3]; however, in some cases, the source of exposure to HPAI H5N1 virus is unknown [35], [36]. Although direct contact with infected sick or dead poultry is the most common risk factor [3], it is likely that inhalation of aerosolized HPAI H5N1 virus is the most likely mode of transmission to infected cases. Some studies have suggested that environmental exposures may also be a risk factor for HPAI H5N1 virus infection. Studies found that lack of indoor water sources was significantly associated with HPAI H5N1 virus infection in Vietnam [37] and in Thailand [38], and H5N1 viral RNA was frequently detected in dust, mud and soil samples collected at farms [39], [40]. Bathing/swimming in contaminated water was associated with HPAI H5N1 virus infection in Vietnam [41] and Cambodia [4], [25].

For the two seropositive household contacts, we believe that the most likely source of HPAI H5N1 virus infection is limited, non-sustained human-to-human transmission. Probable limited non-sustained human-to-human HPAI H5N1 virus transmission has been reported in several case clusters [6], [16], [42], [43]. Neither of the seropositive contacts reported direct contact with sick or dead poultry, or had visited a live poultry market, the main risk factors for HPAI H5N1 virus infection in China [44]. However, both had documented prolonged, unprotected close exposure (i.e. days of providing care or sleeping together) with a symptomatic H5N1 index case, which may have resulted in human-to-human transmission of HPAI H5N1 virus. Since backyard poultry died at the homes of both seropositive contacts, and only a single convalescent serum specimen was collected from each seropositive contact, we cannot completely exclude the possibility of indirect poultry exposure or environmental exposures as the source of HPAI H5N1 virus transmission, either prior to or after the index cases’ illnesses.

Notably, both seropositive household contacts were blood-related family members of an H5N1 index case in the household. Nearly all clusters of H5N1 cases have occurred among blood-related family members, whether linked to a common poultry exposure or where limited, non-sustained human-to-human transmission likely occurred [6], [16], [42], [43]. In one sero-epidemiological study of villagers exposed to poultry, three of seven HPAI H5N1 virus antibody seropositive individuals were blood-related children who lived in different households, and were either asymptomatic or experienced mild illness [4]. Our findings are consistent with observations in other study that have hypothesized a potential genetic susceptibility to HPAI H5N1 virus infection [45]. Further investigations exploring the potential for host genetic risk factors are needed.

Our study is subject to several limitations. Of the close contacts identified, 20% did not agree to participate in the study, and we did not enroll children younger than one year old. Paired sera were not available from all close contacts during the epidemiological investigations. It is possible that we may have missed detection of HPAI H5N1 virus antibodies for some patients with single serum specimens collected <21 days after the last known exposure to an H5N1 case. Nasopharyngeal or throat swab collection combined with serum sample collection among close contacts of H5N1 cases during an outbreak investigation is of paramount importance to assess the extent of transmission and denominator of infected persons among the exposed, to assess the modes of transmission, including the risk of human-to-human transmission, and to assess the spectrum of illness with HPAI H5N1 virus infection. However, without positive viral detection, conclusions based upon a single serum specimen (without a baseline serum specimen or a subsequent convalescent serum specimen to document sero-conversion) may be challenging if serum is collected too soon, or too long after the exposure occurred, as shown in a study of the kinetics of the HPI H5N1 virus neutralizing antibody response, including relatively lower titers in asymptomatic and mildly ill cases compared to severely ill and fatal cases [17]. Strengths of our study are that we used two serologic assays to identify evidence of HPAI H5N1 virus infection as recommended by WHO, and we absorbed out cross-reactive antibodies to seasonal influenza A viruses to increase the likelihood of detecting antibodies to HPAI H5N1 virus. Nevertheless, in testing serum by MN assay from contacts that had HI titers below the screening cutoff to define a seropositive result (≥40), one child contact with an HI titer of 20 also had an MN titer of 40. While we did not consider this child to be seropositive by both serologic assays, this suggests that some asymptomatic or mildly ill children may not mount a robust antibody response to HPAI H5N1 virus infection, and that further data are needed to define the appropriate antibody thresholds to define seropositive results. Another possibility is that the MN assay detected neutralizing antibodies that do not interfere with sialic acid binding as measured by the HI assay.

For future sero-epidemiological investigations, we recommend a standardized approach for sero-epidemiological studies, similar to what was recently suggested for pandemic influenza: (1) systematic sero-epidemiologic investigations following identification of an index patient or an epizootic; (2) standardized data collection to allow pooled analyses worldwide; (3) detailed exposure history (timing and intensity of exposures); (4) standardized laboratory protocols for HI and MN assays, including cutoff titers to define seropositive results; and (5) IRB pre-clearance for all studies or following identification of an outbreak. Ideally, pre-approved protocols will facilitate investigations of and integrated analyses of cohorts of clusters in which human-to-human transmission likely occurred, including collection of monocytes to explore host genetic susceptibility factors. Furthermore, prospective serial collection of sera will allow for better understanding of the kinetics of the antibody response to HPAI H5N1 virus infection, including persons identified with asymptomatic, mild, and severe illness.

In conclusion, we found low seroprevalence of HPAI H5N1 virus antibodies among close contacts of H5N1 index cases. This suggests that during 2005–2008, transmissibility of HPAI H5N1 viruses to and among exposed persons in China was low, even with prolonged, close unprotected exposure to symptomatic H5N1 cases. However, as H5N1 viruses continue to circulate and evolve, the risk of human-to-human transmission of H5N1 viruses is unpredictable and could increase in the future. Seroepidemiologic investigations among exposed individuals should be an ongoing monitoring tool to assess whether HPAI H5N1 viruses circulating among poultry may be adapting to transmit more efficiently to and among people [10], [11]. The ongoing epizootic of HPAI H5N1 virus among poultry in mainland China and elsewhere represents an opportunity to fully assess the risk of poultry-to-human and human-to-human transmission.

## Supporting Information

Table S1 (DOCX)
