# Supplementary material for: Seroprevalence of Antibodies to Highly Pathogenic Avian Influenza A (H5N1) Virus among Close Contacts Exposed to H5N1 Cases, China, 2005–2008
Source: PLoS One. 2013 Aug 13;8(8):e71765. doi: 10.1371/journal.pone.0071765 (PMC3742513; doi:10.1371/journal.pone.0071765)
Supplement: Table S1 — (DOCX) [file pone.0071765.s001.docx]

**Supplementary Table 1: H5N1 serological testing by hemagglutinin inhibition (HI) assay for 231 close contacts exposed to HPAI H5N1 case-patients with paired acute and convalescent sera, China, 2005-2008**

| HI antibody titer of acute sera | HI antibody titer of [convalescent](app:ds:convalescent) sera | | | | |
| --- | --- | --- | --- | --- | --- |
|  | 5^＃^  n (%) | 10  n (%) | 20  n (%) | 40  n (%) | 80  n (%) |
| 5^＃^ | 184 (79.7) | 7 (3.0) | 4 (1.7) | 5 (2.2) | 2 (0.9) |
| 10 | 5 (2.2) | 1 (0.4) | 1 (0.4) | 1 (0.4) | 0 (0) |
| 20 | 1 (0.4) | 0 (0) | 0 (0) | 1 (0.4) | 0 (0) |
| 40 | 12 (5.2) | 0 (0) | 0 (0) | 0 (0) | 2 (0.9) |
| 80 | 4 (1.7) | 0 (0) | 0 (0) | 0 (0) | 1 (0.4) |

Data are no. (%) of 231 close contacts with paired acute and convalescent sera.

^＃^Undetectable titers of <10 are expressed as 5.
